# Is Microsporidial keratitis an emerging cause of stromal keratitis? – a case series study

**DOI:** 10.1186/1471-2415-5-19

**Published:** 2005-08-17

**Authors:** Geeta K Vemuganti, Prashant Garg, Savitri Sharma, Joveeta Joseph, Usha Gopinathan, Shashi Singh

**Affiliations:** 1 Ophthalmic Pathology Service, L.V. Prasad Eye Institute, Hyderabad, India; 2 Cornea Service, L.V. Prasad Eye Institute, Hyderabad, India; 3 Jhaveri Microbiology Centre, L.V. Prasad Eye Institute, Hyderabad, India; 4 Centre for Cellular and Molecular Biology, Hyderabad, India

## Abstract

**Background:**

Microsporidial keratitis is a rare cause of stromal keratitis. We present a series of five cases of microsporidial keratitis from a single centre in southern India with microbiologic and histopathologic features.

**Case presentation:**

Patient charts of five cases of microsporidial stromal keratitis diagnosed between January 2002 and June 2004 were reviewed retrospectively for clinical data, microbiologic and histopathologic data. The presence of microsporidia was confirmed by special stains on corneal scrapings and/or corneal tissues, and electron microscopy. All patients were immunocompetent with a preceding history of trauma in three. Four patients presented with unilateral, small, persisting deep stromal infiltrates, of uncertain etiology, in the cornea, which were not responding to conventional antimicrobial treatment and required penetrating keratoplasty in three. Fifth case was unsuspected and underwent keratoplasty for post-traumatic scar. Three of five cases were diagnosed on corneal scrapings, prior to keratoplasty, while two were diagnosed only on histology. The microsporidia appeared as oval well defined bodies with dense staining at one pole. None of the patients showed recurrence following keratoplasty.

**Conclusion:**

Microsporidia, though rare, should be suspected in chronic culture-negative stromal keratitis. Organisms could lie dormant without associated inflammation.

## Background

Microsporidia are small, oval, obligate intracellular eukaryotic protozoan parasites, widely distributed in vertebrates and invertebrates, but mostly cause infections in immunocompromised individuals [1-3]. The ocular manifestations include superficial punctate keratoconjunctivitis [4-6], and corneal stromal keratitis [7-14] and these two manifestations are directed by the genus involved as well as the immune status of the patient. Keratoconjunctivitis is usually seen in immunocompromised individuals or in contact lens wearers; mostly by genus *Encephalitozoon *while the stromal keratitis is caused by *Nosema *and *Microsporidium*. However, recent reports suggest that keratoconjunctivitis can also occur in immunocompetent individuals [15,16]. A review of the literature in English using PubMed revealed eight cases of corneal stromal involvement, all of which were diagnosed based on identification of the organisms in the tissue sections and/or by ultrastructural studies [7-13]. We present a series of five cases of corneal stromal keratitis from a single tertiary eye care centre, caused by *Microsporidium*, two of which were diagnosed based on the clinical and microbiologic findings. These cases highlight the importance of identifying the clinical presentations, microbiology, and histopathology including electron microscopic findings for proper management of these cases.

## Methods

In a retrospective study, all cases of microsporidial keratitis, diagnosed and managed at L V Prasad Eye Institute, Hyderabad, India between January 2002 and July 2002 were included. The medical records of the patients were specifically reviewed for past and personal history, clinical findings. The microbiologic work-up of the corneal lesion included 1) corneal scrapings stained by Gram, Giemsa stain and potassium hydroxide- calcofluor white preparation, 1% acid fast stain, 2) culture of the scrapings in culture media to facilitate growth of bacteria, fungi and parasites. In cases that underwent therapeutic penetrating keratoplasty, the corneal button was bisected and submitted to microbiology and histopathology. Routine histology stains and special stains like 1% acid fast stain, modified trichrome stain, Gram and Gomori's methenamine silver stain were performed on the permanent sections. In three cases, part of the tissue was submitted in 2% glutaraldehyde for electron microscopy. The patients underwent systemic evaluation to rule out any evidence of immunosuppression, HIV testing of the serum by ELISA (after obtaining informed consent) was done in all cases.

## Case presentation

In this study period, five cases of microsporidial keratitis were diagnosed at L V Prasad Eye Institute, Hyderabad, India. The mean age of patients was 34 (range 2–70 years) years with three male patients. Four cases presented as keratitis, two of which were diagnosed by microbiological processing of the corneal scrapings and two by tissue diagnosis, while one presented with a corneal scar with a post traumatic descemetocele with unsuspected infection. Brief clinical history of all cases (Table 1) is given below.

### Case 1

**Figure 1:**
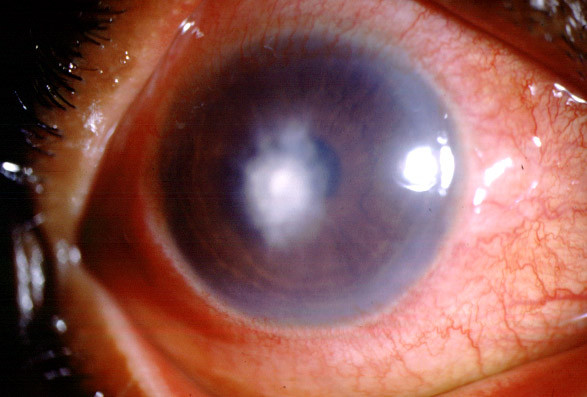
Slit lamp biomicroscopy of cornea of case 1 in diffuse illumination with central mid to deep stromal infiltrate and surrounding stromal edema.

A 40-year-old lady presented to our institute with recurrent pain, redness, watering and blurred vision of seven months duration. During this time she had received topical steroids, antiviral and antifungal medications. However, there was no resolution in the signs or symptoms. The treating surgeon felt she was worsening and referred her as a case of crystalline keratopathy due to the nature of the stromal infiltrate. At presentation, she had a BCVA of perception of light and accurate projection of rays. There was 1+ lid edema and conjunctival congestion. There was a central mid to deep stromal infiltrate measuring 1.5 × 1.5 mm with surrounding stromal edema (figure 1). The overlying epithelium was intact with epithelial edema. Endothelium showed exudates arranged as a sheet over an area of 5.0 × 5.0 mm. There was 360° of superficial vascularisation. Since the infiltrate had not shown any signs of resolution and there was no definitive diagnosis in hand, all the medications were discontinued and she underwent a penetrating keratoplasty for that eye on 25/03/02. The post-operative period was uneventful and at last follow up she was doing well with a BCVA of 6/18. She was treated with topical Chloramphenicol, Prednisolone acetate1%, and Lacrigel. She was later prescribed Tablet Itraconazole 100 mg BD for one month. Her immune status was found to be normal.

### Case 2

Parents of a 2-year-old male child brought him with complaints of white spot on the black of the left eye and swelling of the lids since one and half months. The child had red eye three months back which subsided with eyedrops (Ofloxacin and Tobramycin) but the opacity persisted. Vision could not be assessed as he was photophobic. Examination under anaesthesia revealed flat lids and a quiet conjunctiva. The cornea showed the presence of a mid to deep stromal infiltrate 1.0 × 1.0 mm with surrounding cellular reaction. There was surrounding stromal edema extending about 2 mm from the infiltrate edges. AC was deep and quiet. The IOP was within normal limits. A diagnosis of resolving infectious keratitis or a HSV stromal immune keratitis was kept in mind and he was started on Cefazolin eye drops 2 hourly with close follow up. He showed improvement and 15 days later four hourly Betamet e/d was added. The child was comfortable. He returned two months later with a recurrence of the stromal infiltration in the same location. Deep corneal scrapings were done under anaesthesia which revealed spores suggestive of *Microsporidium*. He was started on oral Itraconazole 50 mg BD. After 15 days of therapy there was no response and considering the risk of amblyopia a penetrating keratoplasty was done on 8/04/02. The post operative period was uneventful and the child is doing well and is relieved of his symptoms as noticed by his parents.

### Case 3

A 23 years male patient presented with a history of pulling sensation in the left eye off and on since 2 years. This was associated with pain, redness and watering. He had been diagnosed and treated as HSV keratitis for the same. He was presently on treatment with Acyclovir ointment 3 times a day and Tears plus eye drops 3 times a day. His visual acuity was counting fingers close to face. The lids were edematous with a diffuse papillary reaction of the conjunctiva. Cornea showed the presence of a central, irregular epithelial defect 2.5 × 3.0 mm with surrounding stromal edema. There was scarring at the edges and the corneal sensations were decreased. Rest of the findings was within normal limits. Viral scrapings were taken and he was started on Acyclovir eye ointment 5 times a day with cycloplegics. The scrapings were inconclusive for viral etiology, there was a clinical resolution and as the epithelial defect reduced in size, he was started on Betamet eye drops drop to drop with the antiviral. The lesion completely resolved and he was continued on topical lubricants.

**Figure 2:**
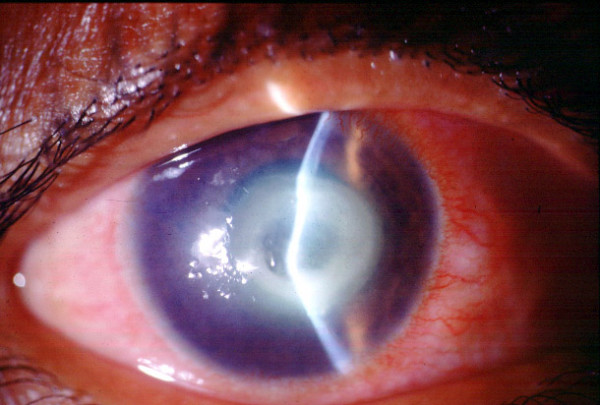
Slit lamp biomicroscopy of cornea of case 3 in diffuse illumination shows a large stromal infiltrate.

He returned with a recurrence 15 days later and was again started on topical corticosteroid under antiviral cover. Oral Acyclovir was also started in a dosage of 400 mg 5 times day. The lesion again resolved and the antivirals were discontinued. However, the lubricant eye drops were continued and the corticosteroid was tapered. He was on regular follow-up and seven months later a recurrence was seen with a stromal infiltrate and the presence of endothelial exudates (figure 2). There were deep vessels seen in the inferior half of the cornea. The central area showed marked thinning and a tissue adhesive with bandage contact lens was applied. The corneal scrapings in Gram stain revealed oval bodies suggestive of microsporidial spores. He was started on oral Itraconazole 100 mg BD with topical ciprofloxacin and cycloplegics. Despite treatment he showed a shallowing of the AC suggestive of leak under the glue and the procedure was repeated. He developed 360° deep and superficial vascularisation. He was continued on the same treatment and is on regular follow-up. On the last follow-up visit the glue and BCL had come off and revealed a scar with thinning of the corneas in that region. He was advised to continue oral therapy for 2 weeks, and come for review.

### Case 4

**Figure 3:**
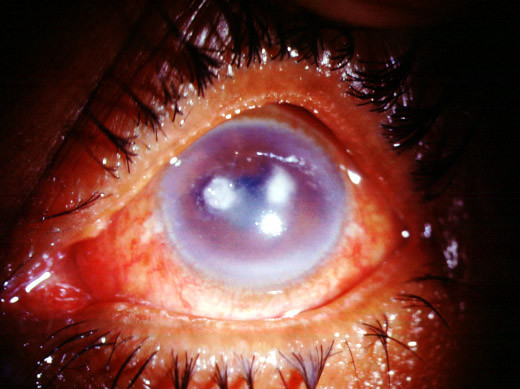
Slit lamp biomicroscopy of cornea of case 4 in diffuse illumination shows two discreet stromal infiltrates.

A 70-year old female presented with complaints of pain, redness, watering and diminution of vision in the left eye of one month duration. She gave history of injury with grass six months ago. Visual acuity in right eye was 6/12 and hand movements with counting finger in the left eye. Right eye on examination was within normal limits. Left eye lids were edematous, conjunctiva was congested, cornea showed the presence of an anterior mid stromal infiltrate in nasal paracentral area with overlying epithelial defect of 1.5 × 1.5 mm (figure 3). In the temporal paracentral area, there was an area of deep stromal infiltrate with underlying endothelial exudate. Smears revealed gram-negative oval bodies. Cultures were positive for gram-positive cocci and gram-positive bacilli. Patient was started on Cefazolin eye drops half hourly. The clinical picture was the same at one and half months, therefore a decision was taken to penetrating keratoplasty in the left eye. Post-operative period was uneventful.

### Case 5

A 37-year-old male presented with history of injury in left eye during a fight 4 years ago. His vision decreased but he did not use any medications. On examination, vision in right eye was 6/6 and in the left eye it was PL positive, PR accurate. Right eye examination was within normal limits. Left eye showed trace conjunctival congestion, there was diffuse corneal scarring with central descemetocoele. Anterior chamber view was hazy and there was total cataract. He underwent optical penetrating keratoplasty with extracapsular cataract extraction with intraocular lens implantation under local anesthesia in left eye. The post-operative period was uneventful and at last follow-up his vision in the left eye was 20/400.

#### Microbiology

**Figure 4:**
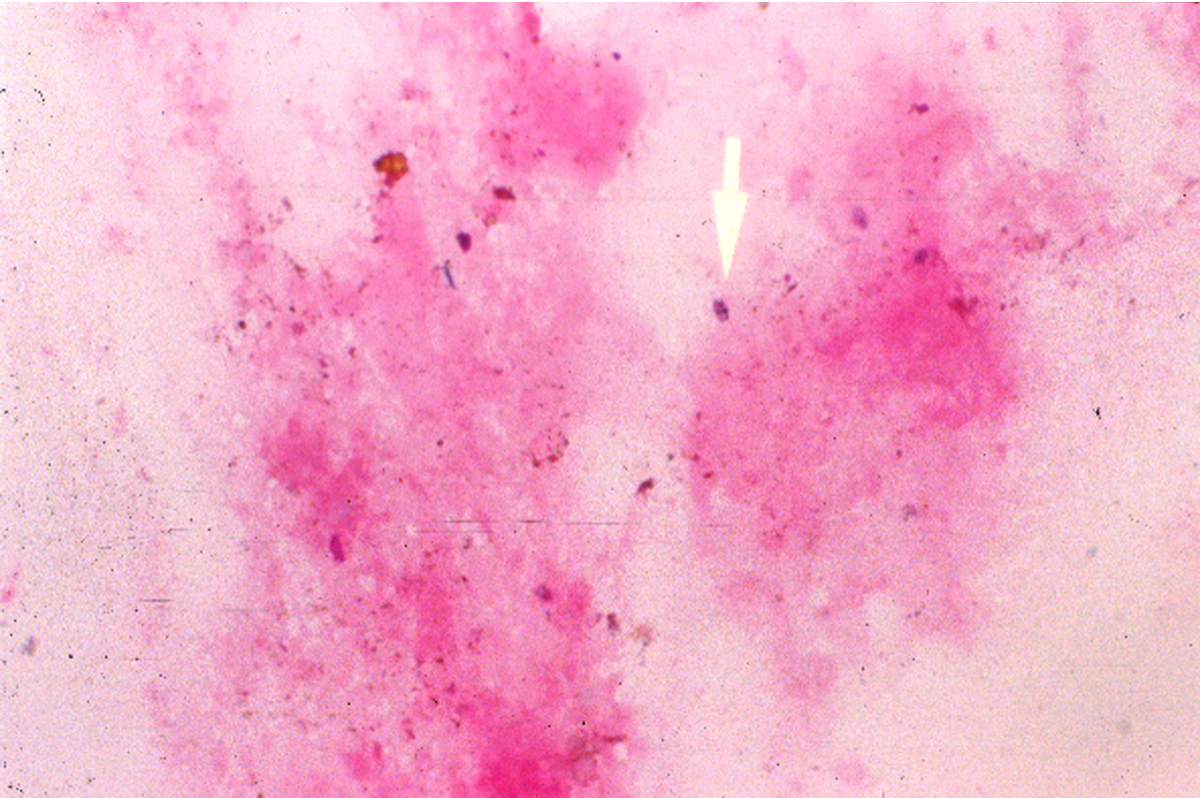
Gram stain of corneal scraping showing gram positive oval microsporidial spores (×500, 4a).

**Figure 5:**
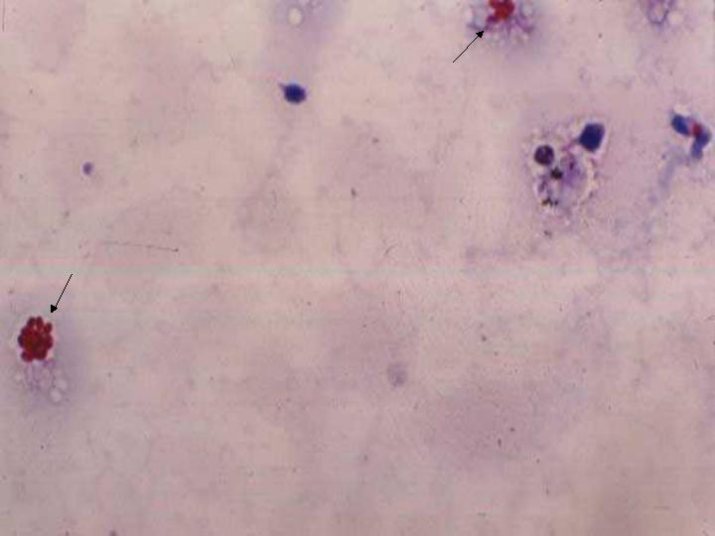
1% acid fast stain of corneal scraping showing acid fast positive oval microsporidial spores (×500, 4b).

Four patients in this series had presented as ulcerative keratitis and one as post-traumatic corneal ectasia. Two of the four patients, diagnosed as stromal keratitis, underwent deep stromal scrapings, which demonstrated microsporidial spores. The diagnosis was made on observing oval refractile bodies in the corneal scrapings, both within cells as well as in extracellular location. The Gram (figure 4) and Giemsa stained smears revealed variable staining of the parasites. The oval body had a dark polar nucleus. The parasite was acid fast and appeared as red oval structure on smears stained with 1% acid fast stain (figure 5). Corneal scrapings stained with calcofluor white showed oval fluorescent bodies in clumps. Bacterial and fungal cultures from these cases did not reveal any organisms except in one case (case 4) that showed a significant growth of *Staphylococcus epidermidis*.

#### Treatment

Three cases were initially treated medically based on the findings of smear examination. In case 1 the treatment was started with fortified cefazolin and fortified gentamicin even though oval bodies were seen in the smear examination. In other two cases where scraping was performed the treatment was started with oral itraconazole 100 mg twice daily and topical ciprofloxacin 0.3%. The infiltrate resolved with medical therapy in one case. Even this case developed progressive thinning for which tissue adhesive and bandage contact lens was applied. Penetrating keratoplasty was performed in four cases. The indications were non-responding keratitis in three cases and thinning with ectasia in one case. Postoperatively all cases were managed with corticosteroids (Prednisolone acetate 1%). The graft was clear in all cases with no evidence of recurrence of infection.

### Histopathology

**Figure 6:**
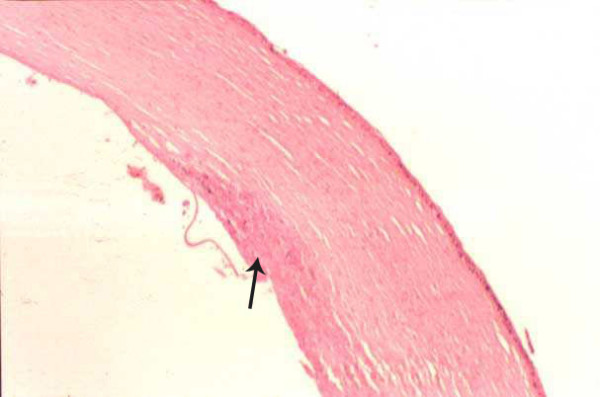
Section showing intact epithelium, with deep stromal infiltrates (arrow) (Hematoxylin and Eosin stain, ×20).

**Figure 7:**
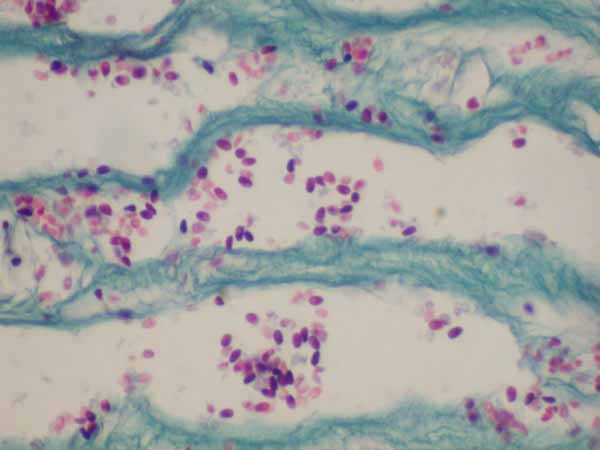
Section of corneal button which shows oval bluish purple spores of microsporidia with a dark band (modified grams chromotrope stain, ×1000).

**Figure 8:**
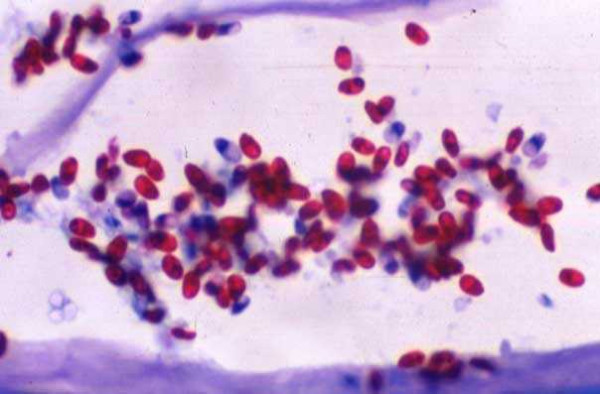
Section of corneal button in which the spores appear red with darkly stained band at the tip (1% acid-fast stain, ×1000).

**Figure 9:**
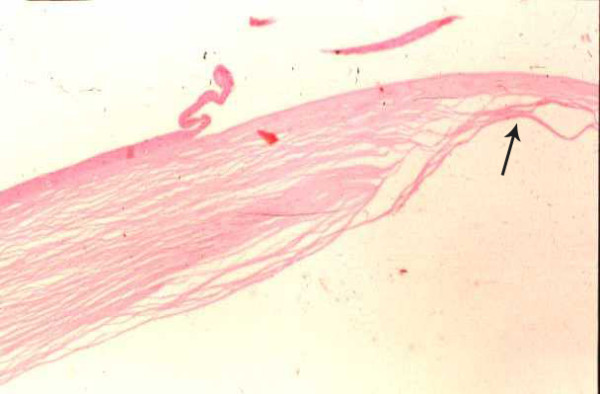
Section of corneal button of case 4 showing marked thinning in the central stroma (descematocele, arrow).

**Figure 10:**
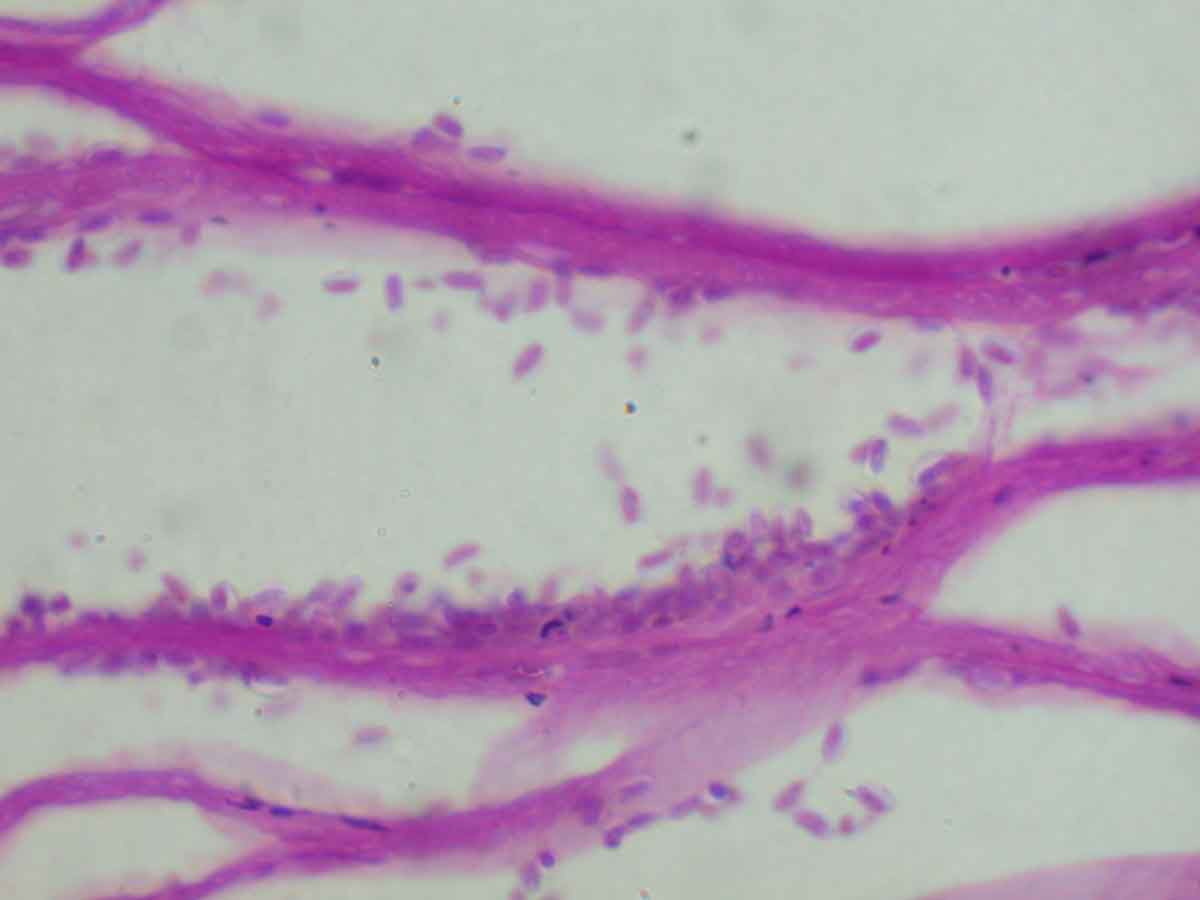
Section of corneal button of case 4 showing the presence of oval, spores of microsporidia in the stroma, unaccompanied by inflammatory infiltrates (PAS stain, ×500).

**Figure 11:**
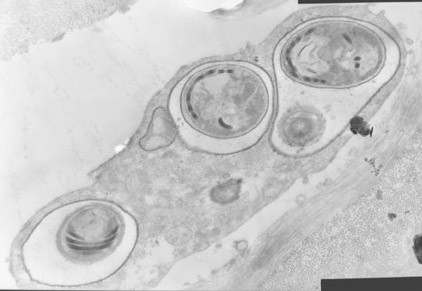
The electron microscopic examination of the corneal button revealing macrophage with three spores.

**Figure 12:**
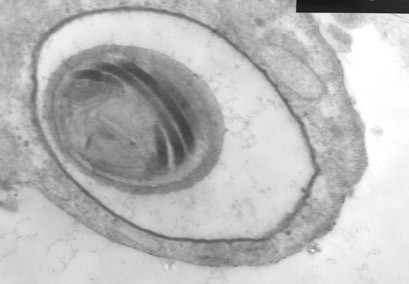
Inset shows an electron lucent thick capsule with a single nucleus and 11 – 13 tubules in the cytoplasm.

Out of four corneal buttons included in the study three were from patients diagnosed as stromal keratitis and one was from a corneal scar with descemetocele, undergoing optical PK. The epithelium was intact in two, showed edema in one and was ulcerated in one case. Bowmans layer was destroyed in 3 out of 4 cases. There was moderate to severe stromal inflammation, at places forming micro abscess (fig 6). The stromal inflammation consisted of polymorphonuclear cells, mostly seen in the pre-Descemet region and extending to the superficial layers. A few macrophages were also seen. Inflammation was absent in the corneal scar tissue. Routine staining showed *Microsporidial spores *as oval bodies, 2 – 3 μ wide and 3 – 5 μ in length involving mostly the deep stroma, with extension into anterior layers in 2 cases. The spores showed a thick band like nucleus at one pole and stained positively with modified Gram Trichrome stain (figure 7), 1% acid fast stain (figure 8) and variably with GMS. The unstained and faintly stained spores showed thick walled capsule that was birefringent on polarized light. The case with corneal scar and a descematocele showed a corneal scar with marked thinning in the central stroma (figure 9). There was no inflammatory infiltrate, but the stroma showed oval, spores of microsporidia in all section (figure 10). These were seen mostly in the deeper stroma, at places extending to mid and superifical stroma. The semithin sections of these tissues showed many viable, mature, immature, and degenerated spores, many of them being intracellular. The electron microscopic examination revealed an electron lucent thick capsule with a single nucleus and 11 – 13 tubules in the cytoplasm (figure 11 with inset as figure 12). Some of the sporoblasts were uniformly dark and osmophilic.

## Discussion

Microsporidia are eukaryotic, spore forming, obligate intracellular, protozoan parasites with two developmental phases – schizogenic and sporogenic. The size of the spores varies from 1 to 20 μm and they are spherical, oval or elongate. They can infect a broad range of vertebrates and invertebrates and are considered ubiquitous [17]. They are becoming increasingly recognized as opportunistic infectious pathogens in immuncompromised patients, causing intestinal, ocular, sinus, pulmonary, muscular and renal diseases [1-3]. Like in all other organs, ocular microsporidial infection was rare before the era of AIDS, the first case being reported by Ashton et al in 1973 [7], in a vascularized corneal scar, unsuspected of infectious etiology. Since 1991, cases of microsporidial keratoconjunctivitis have been reported from immunocompromised patients and of late even in immunocompetent individuals [4-15], one of which was from this centre [16].

Diagnosis of microsporidiosis currently depends on morphological demonstration of the organisms in one or more readily obtainable specimens such as stool, duodenal aspirates, urine, sputum, nasal discharge, bronchoalveolar lavage fluid, conjunctival smears [18]. Definitive species identification is made by using the specific fluorescein-tagged antibody (immunofluorescence) technique, electron microscopy and also by PCR [18-21].

Ocular microsporidia could be isolated or part of systemic microsporidiosis in immunocompromised patients and can manifest as either stromal keratitis or keratoconjuncitivitis. A careful review of previously published cases and the cases reported in this series suggests that microsporidial stromal keratitis is a slowly progressive keratitis-affecting individual of any age. No definitive predisposing factors could be identified in this series. The history of trauma was elicited in only 2 of 5 cases from our series and 1 of 7 previously published reports [7].

Clinically, microsporidial stromal keratitis could mimic suppurative or non suppurative inflammation and vascularization of the cornea and includes a differential diagnosis of herpes simplex virus keratitis, fungal or bacterial keratitis. The diagnosis can be made either by corneal biopsy or deep corneal scrapings as was done in two of our cases. One of the non-invasive emerging technique of diagnosing microsporidial keratitis is with the help of a confocal microscopy [21] using a 24× contact objective lens and a Nipkow disc in imaging. The spores appear as high contrast intraepithelial or intrastromal opacities.

The duration of symptoms in this series ranged from one month to 2 years suggesting a slow indolent nature in the initial phase. Except one case, none of them gave any history of preceding trauma, illness, immunosupression or any local predisposing factors. The youngest patient in this series was 2 years, which is very unusual. Four of the cases in this series were diagnosed as infectious keratitis; two were diagnosed and treated as viral keratitis to which they showed response but after few months showed recurrence of infiltrates. The subsequent corneal scrapings in three cases were diagnosed as microsporidial keraitits. This is in contrast to the other reports where the diagnosis was made only on corneal sections.

On microbiologic examination of the corneal scraping, the differential diagnosis of microsporidia includes bacteria or yeast, but can be confirmed by special stains like Grams trichrome stain, 1% acid fast stain [19,22-24]. These oval or round structures are non-budding which differentiates them from yeast. Histologically, the organisms are seen within the stromal keratocytes, histocytes as well as between the stromal lamellae. They are seen as oval gram positive structures with a thick wall. They stain red with modified trichrome stain and acid fast stain best demonstrates the organisms. However, it should be noted that all species may not be acid fast. The inflammatory cells in the corneal stroma consisted of polymophonuclear cells as well as few histiocytes, with varying degrees of stromal necrosis, thus explains the corneal thinning in these cases.

Under electron microscope the spores of microsporidia show sporoplasm and a tubular polar filament with varying number of coils depending on the species. Therefore, ultrastructurally, it is possible to identify the species. The organisms of *Nosema *species, as seen in our series, measure 3 – 5 microns in length and 2 – 3 micron in width. The organisms are not surrounded by parasitoporous vacuole within the host cells and the number of coils in the cytoplasm varies between 10 – 14.

Therapeutically, there is no definitive medical therapy for this entity. Font et al treated their case with fumagillin 0.3% and oral Albendazole but there was no response. After making a diagnosis of microsporidial keratitis in our series, two received itraconazole for few weeks, while one case received cefazolin. Only one case responded to itraconazole treatment while the other two cases showed persistence of infiltrates with corneal thinning, and necessitated surgical intervention. Even in the case that responded to medical treatment there was progressive thinning that required application of tissue adhesive. Though this is a small case series, but reviewing the literature and in the light of these findings, it is possible for us to suggest that in the absence of effective medical therapy it is reasonable to manage these cases surgically. Penetrating keratoplasty helped eradicating infection in 4 cases of our series and 6 cases from previously published reports. It is important to note that the infection is mostly restricted to stroma and has not been demonstrated in deeper ocular tissues. The other favorable feature in these cases is that none of them had recurrence of infection postoperatively.

It is not clear how the microsproidia enter into the eye- it could be either due to trauma or contact with contaminated water, food. The normal lifecycle of microsporidia includes: once invasion of the spore into the human host cell occurs, the contents are discharged into the cytoplasm. Within the cell the sporoplast divides by binary fission to form schizont with 2–6 nuclei, which split into unicellular meronts. The meronts then secrete a rigid capsule and the fully formed spore measures about 2.5 × 1.5 microns. The cell eventually ruptures to continue the cycle and further destruction of the host tissue.

## Conclusion

This is the largest series of ocular infection by *Microsporidia *in immunocompetent individuals manifesting as stromal keratitis. This series suggests the need for a high index of suspicion in diagnosing microsporidial infection in culture negative stromal keratitis. Use of special stains, tissue biopsy and electron microscopy aids in confirming the diagnosis of this infection. Due to lack of effective medical therapy, surgical treatment appears to be a reasonable option in the management of these cases.

## Competing interests

The author(s) declare that they have no competing interests.

## Authors' contributions

GKV was involved in collecting data, carrying out the analysis and drafting the manuscript. PG provided the clinical information and edited the manuscript. SS participated in its design, carried out the microbiological work up and edited the manuscript. JJ collected data, participated in its design and helped to draft the manuscript. UG participated in its design and carried out the microbiological work up. Ssingh carried out the electron microscopic analysis. All authors read and approved the final manuscript.

## Pre-publication history

The pre-publication history for this paper can be accessed here:



## Figures and Tables

**Table 1:** Summary of clinical features in Microsporidial keratitis

Age/	Dur of Sym	Med. Tx	trauma	vision	Infiltrate	Vasc	Clin diag	Micro	Treat	FU
40/F	7 mon	AV, AF	-	6/8		+	Crys K	Neg	-	PK
2/F	1.5 mon	AB	-				HSV	Micros	Itracon	PK
23/M	2 yrs	AV	-	cfcf		+	HSV	Micros	Itracon	Scar
70/F	1 mon	?	-	cfcf			IK	Micros	Cefaz	PK
37/F	4 mon	-	+				Desce	Neg	Nil	PK

AB = antibacterial

AV = antiviral

AF = antifungal

Cfc = counting fingers close to face

HSV = herpes simplex keratitis

Crys K = crystalline keratopathy

PK = Penetrating Keratoplasty

Micros = Microsporidia
